# An Innovative Approach to Enhancing the Surveillance Capacity of State-based Diabetes Prevention and Control Programs: The Diabetes Indicators and Data Sources Internet Tool (DIDIT)

**Published:** 2005-06-15

**Authors:** Qaiser Mukhtar, Erica R Brody, Prachi Mehta, Jenny Camponeschi, Cynthia K Clark, Jay Desai, Michael Friedrichs, Angela M Kemple, Heidi R Krapfl, Brenda Ralls, Jackson P Sekhobo

**Affiliations:** Division of Diabetes Translation, Centers for Disease Control and Prevention (CDC); RTI International, Research Triangle Park, NC; Northrop Grumman CITS Contract, Atlanta, Ga; Wisconsin Department of Health and Family Services, Division of Public Health, Diabetes Prevention and Control Program (DPCP), Madison, Wis; CDC, Atlanta, Ga; Minnesota Diabetes Program, Minnesota Department of Health, St. Paul, Minn; Utah Bureau of Health Promotion, Department of Health, Salt Lake City, Utah; Oregon Department of Human Services, DPCP, Portland, Ore; New Mexico Diabetes Control Program, Department of Health, Santa Fe, NM; Utah DPCP, Department of Health, Salt Lake City, Utah; Diabetes Surveillance and Evaluation, Bureau of Chronic Disease Epidemiology and Surveillance, New York State Department of Health, New York, NY

## Abstract

The Diabetes Indicators and Data Sources Internet Tool (DIDIT) is an interactive Web-based resource with information on 38 diabetes indicators (e.g., diabetes-associated complications, care, lifestyle) and 12 associated data sources frequently used by state diabetes prevention and control programs. This tool is designed to strengthen the ability of states to conduct diabetes surveillance and to promote consistency in defining and tracking indicators across states. In this way, the DIDIT supports one of the 10 essential public health services: the timely and accurate assessment of public health.

In addition to serving as a central repository of information on diabetes surveillance, the DIDIT also allows users to share experiences of using these indicators and data sources in their diabetes surveillance activities, data analysis, and tracking of diabetes-related objectives stated by *Healthy People 2010*. The DIDIT is an innovative approach to enhancing public health surveillance at the state and national levels.

## Introduction

Diabetes is a complex, chronic disease consisting of a group of conditions resulting from the impaired production or effectiveness of insulin (1). It is increasingly important to conduct surveillance to accurately assess the prevalence of diabetes and its complications. The global prevalence of diabetes is nearly 200 million cases, and there is a sense of urgency in the public health field about understanding and defining the burden of diabetes and its associated risk factors (2). Traditionally, diabetes surveillance has included monitoring indicators such as incidence, prevalence, complications, mortality, quality of care, and preventive-care practices (3). Reflecting recent developments in the identification of risk factors for type 2 diabetes (4,5), surveillance has expanded to include lifestyle variables such as diet and physical activity (6), socioeconomic status (7), quality of life (8), and a condition called prediabetes. People who have blood glucose levels that are higher than normal, but not high enough to be classified as diabetes, have prediabetes (4).

The Centers for Disease Control and Prevention's (CDC's) National Diabetes Prevention and Control Program (NDPCP) supports a Diabetes Prevention and Control Program (DPCP) in each state, each U.S. territory, and the District of Columbia. The NDPCP and the 59 DPCPs are charged with defining and monitoring the burden of diabetes and demonstrating success in accomplishing NDPCP national objectives, which were established in 1999 to reduce preventable morbidity and mortality associated with diabetes. These objectives incorporate goals to increase the proportion of people with diabetes who receive annual foot examinations, dilated eye examinations, hemoglobin A1c (HbA1c) tests, and recommended vaccinations against influenza and pneumococcal infection. The national objectives also address reducing health disparities for populations at high risk for diabetes and establishing links with other programs that promote wellness among people with diabetes (10). 

Additionally, the timely and accurate assessment of public health is one of the 10 essential public health services and thus a fundamental function of public health agencies, such as DPCPs, that are responsible for population-based health (11). The DPCPs also track and strive to achieve *Healthy People 2010* objectives, 17 of which directly address diabetes (12).

Surveillance is essential to effective program planning, advocacy, and evaluation, and surveillance data contribute significantly to developing and carrying out policies at the national and state levels. *Surveillance* is the continuous monitoring or routine collection of data on various factors (e.g., behaviors, complications, deaths) over a regular interval of time, and surveillance data can be useful for program evaluation. In contrast, *evaluation* provides tailored information to answer specific questions about a program. Data collection for evaluation is more flexible than for surveillance and may allow specific program areas to be assessed in greater depth (13).

The NDPCP requires each DPCP to establish a diabetes surveillance system to monitor the burden of diabetes within its state and systematically evaluate the impact of DPCP programs on the morbidity and mortality associated with diabetes. The 59 DPCPs differ in their resources, funding levels, expertise, priorities, staffing, and partnerships. These factors affect a program's ability to collect, analyze, and interpret data and to conduct surveillance and evaluate programs. A diabetes-specific centralized resource providing comprehensive background information on definitions of indicators, data sources, methodology, and issues relevant to analysis is important to achieving these surveillance and evaluation goals.

In the fall of 2001, representatives from NDPCP and five of the 59 DPCPs formed a work group that began developing a tool to assist the DPCPs with surveillance and program evaluation. The result was the Diabetes Indicators and Data Sources Internet Tool (DIDIT), which was developed to strengthen the DPCPs' ability to conduct surveillance and program evaluation.

**Figure 1:**
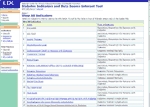
List of all indicators in the Diabetes Indicators and Data Sources Internet Tool (DIDIT) by type.

The DIDIT is an interactive resource that provides comprehensive information on 38 indicators of diabetes and their data sources. Although most of the materials found in the DIDIT are available elsewhere (e.g.,http://www.cdc.gov/diabetes/statistics/index.htm; www.cdc.gov/brfss), the intent of the DIDIT is to offer, in a centralized location, the most recent information on diabetes surveillance. Another goal is to present clear, concise, and easy-to-read information by organizing it into a flexible and an interactive format. In addition to providing information on 38 diabetes indicators (Figure 1) and other associated data sources (if available) for each indicator, the DIDIT provides information such as lists of related articles, Web site links, and recommended standards of care. Updated on an ongoing basis, the DIDIT facilitates communication in the diabetes community by allowing for user feedback and comments about indicators and data sources. In addition, users have the opportunity to share their general experiences with using the tool. In this article, we describe the DIDIT's content, provide examples of how its information is presented, and highlight its public health applications. We provide an overview of the process used to develop the DIDIT; a comprehensive description of the DIDIT development process will be published in a companion article.

## Developing the DIDIT

The DIDIT was developed by the members of a work group representing DPCPs and the NDPCP. The work group members have extensive expertise in diabetes surveillance and evaluation. User input has also been obtained from additional DPCPs through 1) focus groups, 2) usability testing, and 3) pilot testing, which the NDPCP conducted with volunteer DPCPs before the DIDIT's release. Content and Web site development are supported by two independent contractors. To facilitate content development, the work group members developed a list of 55 diabetes indicators and, using two rounds of Delphi process, ranked them based on importance, mutability, and availability of state-level data. In this way, the DIDIT's 38 indicators were selected. The national- and state-level data sources described in the DIDIT were selected because they are publicly available, reliable, and accurate as a result of quality assurance procedures in place at the various agencies that maintain them (e.g., the CDC, the Agency for Healthcare Research and Quality, and the Centers for Medicare and Medicaid.) The first version of the DIDIT was released to DPCPs in October 2003. In April 2004, the DPCP staff and the NDPCP project officers were trained to use the DIDIT using NetMeeting (Microsoft Corp, Redmond, Wash), which allowed for a two-way interaction between stakeholders in various geographical areas. 

The DIDIT is still a work in progress, and several enhancements are underway. The DPCPs and CDC partners have been kept informed of the DIDIT development and enhancement on an ongoing basis through presentations, e-mail updates, and conference calls. 

## Contents of the DIDIT 

### Diabetes indicators

**Figure 2:**
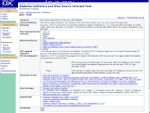
Information in the Diabetes Indicators and Data Sources Internet Tool (DIDIT) on each of 38 diabetes indicators.

The DIDIT provides information about each of 38 diabetes indicators (Figure 2), including its relevance to diabetes surveillance, NDPCP national objectives, and *Healthy People 2010* objectives (12). Additionally, it provides information about the agencies recommending the indicator, related articles and Web sites, and a list of associated data sources. Many of the indicators are related to health care practices (e.g., annual foot examinations) or recommendations on lifestyle issues for people with diabetes (e.g., physical activity). If relevant, information on recommended standards of care is provided. For example, the American Diabetes Association's position statement on preventive care practices is linked to many of the indicators.

### Data sources

**Figure 3:**
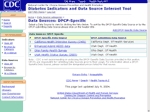
List of Diabetes Prevention and Control Program-specific data sources in the Diabetes Indicators and Data Sources Internet Tool (DIDIT).

**Figure 4:**
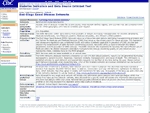
General information about the End Stage Renal Disease Networks data source that is included in the Diabetes Indicators and Data Sources Internet Tool (DIDIT).

The DIDIT includes 12 frequently used state and national data sources for diabetes surveillance (Figure 3). The data sources are cross-referenced with information about the indicators. For each indicator, the DIDIT provides methodologies for computing the numerator and denominator using the associated data sources. In addition, DPCPs can enter and share information about a DPCP-specific data source to inform others of special methods for collecting local data. As of April 2005, California, Utah, and Ohio had entered information about DPCP-specific data sources they have used for diabetes surveillance (Figure 3). The DIDIT data sources include surveys (e.g., Behavioral Risk Factor Surveillance System [BRFSS], National Health Interview Survey [NHIS]), administrative records (e.g., health insurance claims), and vital records (e.g., death and birth certificates). These data are collected by various methods, such as telephone interviews, in-person interviews, and laboratory testing. We should note that the DIDIT does not provide data but instead gives comprehensive information about data, including where and how to access it. For each data source, the DIDIT provides information on the population represented by the data, the method and purpose of data collection, access issues, privacy issues, and general analytic issues, in addition to providing a list of diabetes indicators that can be tracked using that data source (Figure 4). The DIDIT is designed to provide users with the information needed to use a data source effectively. Thus, the DIDIT serves as a reference guide for learning about and using key data sources for diabetes surveillance. The DIDIT also has a search feature, allowing users to search for specific data sources, indicators, or both.

### Specification of numerators and denominators

Standardization in computing and reporting estimates across states is essential to high-quality diabetes surveillance. Any differences in calculating the numerator or denominator of an indicator at the state level can cause variations, making it difficult to compare data over time or across states.

**Figure5:**
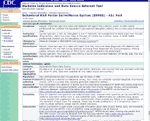
General and diabetes indicator-specific information provided by the Behavioral Risk Factor Surveillance System (BRFSS) data source that is included in the Diabetes Indicators and Data Sources Internet Tool (DIDIT).

The DIDIT provides instructions for constructing numerators and denominators of indicators (Figure 5) for each data source linked to each indicator. For example, state-level data on HbA1c testing is available from sources such as the Health Plan Employer Data and Information Set (HEDIS), BRFSS, Medicare, and Medicaid. The DIDIT provides information about computing the numerator and denominator for HbA1c testing using each of these four data sources. It provides information on which cases should be included in the numerator and denominator in addition to relevant diagnosis and procedure codes where appropriate (e.g., for administrative and inpatient hospital discharge data). For survey data, relevant survey questions are provided (Figure 5), allowing users to analyze and report diabetes indicators consistently with other programs. All of this information allows users to compare different potential data sources and decide which are best suited for their program needs and how to correctly define the numerator and denominator for data analysis. 

### Technical issues in analysis

**Figure6:**
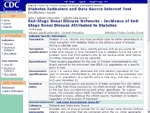
Information to construct the incidence of end-stage renal disease attributed to diabetes indicators using the End Stage Renal Disease Networks data source.

Data analysis can be complex, and most people learn about its pitfalls through trial and error. A unique feature of the DIDIT is the *technical notes* field, which provides background information and caveats for analyzing data for an indicator and a data source (Figure 6).

Furthermore, the DIDIT encourages users to share experiences in analyzing indicators and data sources. By using the comments *view/add* feature (Figure 4) and posting their comments on the DIDIT, users can inform their peers about technical issues related to diabetes data analysis. This interactive feature of the DIDIT sets up an avenue for sharing, documenting, and learning. Currently, all DPCPs can enter this information about their own or their partners' experiences.

### At a Glance

**Figure7:**
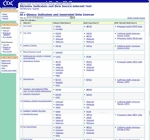
List of diabetes indicators and their associated national-, state-, and Diabetes Prevention and Control Program (DPCP)-specific data sources.

**Figure8:**
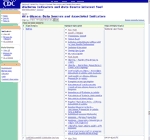
List of Diabetes Indicators and Data Sources Internet Tool (DIDIT) data sources by type and related indicators.

The *At a Glance* function in the DIDIT provides a snapshot of the relationship between indicators and their data sources. For example, in Figure 7, the 38 indicators are presented in the first column, with their national- and state-level data sources presented in the second and third columns. This layout allows users to quickly assess the number and names of data sources available in the DIDIT for each indicator. Similarly, the DIDIT presents another table with the 12 data sources listed in the first column and the indicators that can be measured with each data source in the second column (Figure 8).  

## Upcoming Enhancements and Activities 

In its current capacity, the DIDIT is a powerful tool, but it continues to evolve in response to the needs of the NDPCP and DPCPs. Thus, the DIDIT is a work in progress. While we work on populating the current fields, efforts are underway to enhance the tool by adding a glossary of epidemiology and surveillance terms, links to useful references, and a section called *Resources* that lists background materials frequently used by epidemiologists. We plan to update the DIDIT content as needed and develop an online training curriculum. Additionally, we plan to evaluate the DIDIT to ensure that it is meeting the needs of DPCPs and their partners.

## Using the DIDIT for Program Evaluation Activities 

An additional function of the DIDIT is to assist in evaluating public health programs. The information provided in the DIDIT can address steps 3–5 of the CDC's 6-step framework for program evaluation (14). For example, if a DPCP is planning to evaluate the increase in the percentage of people with diabetes who receive the recommended eye examination, they might use the DIDIT to help them and their community partners understand the reason it is important to track eye examinations among people with diabetes and how this indicator is defined. Next, using information on available data sources, they would identify data sources for tracking eye examinations (e.g., BRFSS, HEDIS) and use the descriptive information about each data source to select the best data source for their purpose. Using information in the *indicator-specific* section, they would construct their numerator and denominator for eye examinations in a way that is consistent with methods used by other DPCPs. The information in *technical notes* would help them analyze the data and interpret their conclusions.

## Implications for Public Health Practice 

Effective public health surveillance requires access to timely, accurate, and reliable information from a wide variety of sources (3). The DIDIT is a valuable resource for helping public health practitioners and clinical staff to assess the burden, complications, and risk factors of diabetes.

The DIDIT is the only reference tool of its kind that contains diabetes-specific information required for surveillance and, to some extent, for program evaluation activities. This comprehensive resource saves time because DIDIT users do not have to search for information on several Web sites or read lengthy descriptions to find information on diabetes.

The information about indicators has numerous potential uses. People new to diabetes surveillance can use the information to become familiar with multiple aspects of each indicator. The information also assists experienced public health practitioners by helping them work with new indicators or stay current on the most recent diabetes information, such as the latest position statement of the American Diabetes Association. Having a list of related peer-reviewed articles, summaries, or Web sites for each indicator saves users the effort of searching the literature. Informally, some users have reported using information and resources from the description pages on various indicators to prepare presentations, write articles, and locate and develop educational materials.

The DIDIT provides a common platform and language for epidemiologists, state program coordinators, the CDC's Division of Diabetes Translation, and project officers to communicate about issues on diabetes surveillance and analysis. In addition, it is designed to promote national uniformity in collecting and defining data and computing diabetes indicators. Finally, it provides a mechanism for DPCPs to share their unique state and local data sources, in addition to their own experiences with using and analyzing diabetes indicators and data sources.

The concepts and participatory processes used for the DIDIT's development were designed to be user friendly and can be applied to many areas of public health practice. They also may be used to develop information technology solutions for other diseases, public health settings, or program areas. The DIDIT serves as a model approach for other local, state, and national public health programs interested in enhancing their and their partners' technical and scientific ability by emphasizing collective experience and information technology. Additionally, the DIDIT provides a template that may be applied to other chronic disease programs at the CDC.

## Access to the DIDIT 

The DIDIT is currently accessible to all NDPCP and DPCP staff through the Diabetes Management Information System. To ensure data integrity and security, this system is password protected on a secure site. Individuals in the diabetes community can request access to the DIDIT by visiting the CDC's Diabetes Public Health Resource Web site (www.cdc.gov/diabetes/statistics/index.htm) and filling out a request form. Since its release in October 2003, more than 30 external partners have requested and been granted access to the DIDIT. 
