# Supplementary material for: An Innovative Approach to Enhancing the Surveillance Capacity of State-based Diabetes Prevention and Control Programs: The Diabetes Indicators and Data Sources Internet Tool (DIDIT)
Source: Prev Chronic Dis. 2005 Jun 15;2(3):A14. (PMC1364523)
Supplement: Supplementary file 1 — View a full-size PDF of Figure 1 (72K) [file 04_0126_01.pdf]

Diabetes Indicators and Data Source Internet Tool

DIDIT
- Home
- Search
- Reports

EPI RESOURCES

Indicators
- View All
- Browse by Category
- At a Glance

Data Sources
- View All
- Browse by Category
- At a Glance

ABOUT INDICATORS

Background
Contact Information

Home »

View All Indicators

Select an Indicator to view by clicking the links below. To sort by the name or type of Indicator please click on the header title.

View All Indicators

#	Name	Type of Indicator
1	A1c Level	Secondary Prevention for Persons with Diabetes
2	A1c Test	Secondary Prevention for Persons with Diabetes
3	Aspirin Therapy	Secondary Prevention for Persons with Diabetes
4	Blood Pressure Level	Secondary Prevention for Persons with Diabetes
5	Cardiovascular Death in Persons with Diabetes	Diabetes-related Mortality
6	Cholesterol Tested	Secondary Prevention for Persons with Diabetes
7	Dental Exam	Secondary Prevention for Persons with Diabetes
8	Diabetes Care Related Office Visit to a Health Professional	Secondary Prevention for Persons with Diabetes
9	Diabetes Education	Diabetes Patient Education
10	Diabetes-related Hospitalizations	Diabetes-related Complications
11	Diabetes-related Mortality	Diabetes-related Mortality
12	Dilated Eye Exam	Secondary Prevention for Persons with Diabetes
13	Flu Vaccination	Secondary Prevention for Persons with Diabetes
14	Foot Exam	Secondary Prevention for Persons with Diabetes
15	Hospitalization for Cardiovascular Disease among Persons with Diabetes	Diabetes-related Complications
16	Hospitalization for Lower Extremity Amputations	Diabetes-related Complications
17	Incidence of End-Stage Renal Disease Attributed to Diabetes	Diabetes-related Complications
18	Incidence of Gestational Diabetes	Prevalence & Incidence of Diabetes
19	LDL-C Level	Secondary Prevention for Persons with Diabetes
20	Monitoring for Diabetic Nephropathy	Secondary Prevention for Persons with Diabetes
21	Obesity - Primary Prevention in Adults	Lifestyle
22	Obesity - Secondary Prevention in Adults with Diabetes	Lifestyle
23	Overweight or Obese - Secondary Prevention in Adults with Diabetes	Lifestyle
24	Overweight or Obese- Primary Prevention in Adults	Lifestyle
25	Pneumococcal Vaccination	Secondary Prevention for Persons with Diabetes
26	Prevalence of Cardiovascular Complications among Persons with Diabetes	Diabetes-related Complications
27	Prevalence of Diabetes in Adults	Prevalence & Incidence of Diabetes
28	Prevalence of Diabetes in Children	Prevalence & Incidence of Diabetes
29	Prevalence of Diabetic Retinopathy	Diabetes-related Complications
30	Prevalence of End-Stage Renal Disease	Diabetes-related Complications
31	Prevalence of Foot Ulcers	Diabetes-related Complications
32	Regular Physical Activity - Primary Prevention in Adults	Lifestyle
33	Regular Physical Activity - Secondary Prevention in Adults with Diabetes	Lifestyle
34	Self-Blood Glucose Monitoring	Diabetes Self-care
35	Smoking - Primary Prevention	Lifestyle
36	Smoking - Secondary Prevention in Adults with Diabetes	Lifestyle
37	Unhealthy Days among Adults with Diabetes	Diabetes-related Complications
38	Visual Foot Exam (self or someone other than health professional)	Diabetes Self-care
