# Supplementary material for: An Innovative Approach to Enhancing the Surveillance Capacity of State-based Diabetes Prevention and Control Programs: The Diabetes Indicators and Data Sources Internet Tool (DIDIT)
Source: Prev Chronic Dis. 2005 Jun 15;2(3):A14. (PMC1364523)
Supplement: Supplementary file 2 — View a full-size PDF of Figure 2 (53K) [file 04_0126_02.pdf]

Diabetes Indicators and Data Source Internet Tool

A1c Test

**Category**	Secondary Prevention for Persons with Diabetes
**General definition**	The percentage of persons with diabetes who have had at least two A1c tests during the past year.
**Rationale**	Glycemic control is essential for preventing diabetes-related complications. A1c exams provide information on blood sugar levels over the prior 2-3 months. This process indicator provides information about the quality of diabetes care provided.
**Data Sources for this Indicator**	• View All<br>• National<br>• State<br>• DPCP-Specific
**Recommended by**	• CDC<br>• Health Disparities Collaboratives*<br>• Indian Health Service*<br>• National Diabetes Quality Improvement Alliance (formerly DQIP)*<br>• NCQA*
**DDT national objective**	This indicator links to the Division of Diabetes Translation (DDT) National Objective to increase the percentage of persons with diagnosed diabetes who receive A1c tests.
**HP 2010 objective**	Objective 5-12: To increase the proportion of adults with diabetes who have at least two glycosylated hemoglobin measurements during the year.<br><br>Baseline: Fifty-nine percent of adults aged 18 years and older with diabetes had at least two glycosylated hemoglobin measurements during the year, (BRFSS, age-adjusted, 2000).<br><br>Target: Fifty percent of adults aged 18 years and older with diabetes having at least two glycosylated hemoglobin measurements during the year.
**Standards of care**	• American Diabetes Association*<br>• National Quality Forum, National Voluntary Consensus Standards for Adult Diabetes Care. Available from: The National Quality Forum, A601 Thirteenth Street, NW, Suite 500 North, Washington, DC 20005; Tel: 202.783.1300.*<br>• Use of Glycated Hemoglobin and Microalbuminuria in the Monitoring of Diabetes Mellitus. Summary, Evidence Report/Technology Assessment: Number 84. AHRQ Publication No. 03-E048, July 2003. Agency for Healthcare Research and Quality, Rockville, MD. *
**Data summary**	• Diabetes Surveillance System<br>• J.B. Saaddine, M.M. Engelgau, G.L. Beckles, E.W. Gregg, T.J. Thompson, and K.M.V. Narayan. (2001). A Diabetes Report Card for the United States: Quality of Care in the 1990s. Annals of Internal Medicine, 136(8): 565-574.<br>• Medicare Health Plan Compare*
**Related links**	• American Diabetes Association: A1c test*<br>• Guide to Community Preventive Services<br>• Healthy People 2010<br>• National Diabetes Education Program - Check Your Hemoglobin A1c I.Q.*<br>• The National Glycohemoglobin Standardization Program*
**Comments**	View<br>Add
