# Supplementary material for: An Innovative Approach to Enhancing the Surveillance Capacity of State-based Diabetes Prevention and Control Programs: The Diabetes Indicators and Data Sources Internet Tool (DIDIT)
Source: Prev Chronic Dis. 2005 Jun 15;2(3):A14. (PMC1364523)
Supplement: Supplementary file 3 — View a full-size PDF of Figure 3 (77K) [file 04_0126_03.pdf]

National Center for Chronic Disease Prevention and Health Promotion

Diabetes Indicators and Data Source Internet Tool

DIDIT
- Home
- Search
- Reports

EPI RESOURCES

Indicators
- View All
- Browse by Category
- At a Glance

Data Sources
- View All
- Browse by Category
- At a Glance

ABOUT INDICATORS

Background
Glossary
User Experiences
Contact Information

Home » Data Source categories »

Data Sources: DPCP-Specific

Select a Data Source to view by clicking the links below. To sort by the DPCP-Specific Data Source or by the DPCP submitting Data Source please click on the header title.

Add DPCP-Specific Data Source

Data Sources: DPCP-Specific

#	DPCP-Specific Data Source	DPCP submitting Data Source
1	California Health Interview Survey (CHIS)	California Dept. Of Health Services
2	Enhanced Hybrid HEDIS data	Utah Dept. Of Health
3	Ohio Family Health Survey	Ohio Dept. Of Health
4	Utah Health Status Survey (2003)	Utah Dept. Of Health

Note - CDC is not recommending or endorsing the DPCP-Specific Data Sources provided in this section. DPCPs are responsible for validating Data Sources prior to their usage. For information about a DPCP-Specific Data Source, please contact the designated person listed for that source.

This page last updated July 9, 2004.
