# Supplementary material for: An Innovative Approach to Enhancing the Surveillance Capacity of State-based Diabetes Prevention and Control Programs: The Diabetes Indicators and Data Sources Internet Tool (DIDIT)
Source: Prev Chronic Dis. 2005 Jun 15;2(3):A14. (PMC1364523)
Supplement: Supplementary file 4 — View a full-size PDF of Figure 4 (58K) [file 04_0126_04.pdf]

Diabetes Indicators and Data Source Internet Tool

End-Stage Renal Disease Networks

**General Information - End-Stage Renal Disease Networks***	
**Category**	National and State Data Source
**Level of geographic aggregation**	Possible units of analysis include the US and states, renal network-defined regions, and counties that are contained within the US. Other geographic units may be available upon request.
**Population**	U.S. Population
**Method of data collection**	The ESRD Networks collect data directly from providers of dialysis and kidney transplantation for services obtained by Medicare beneficiaries, non-Medicare patients, Medicare secondary, and Veteran's Affairs patients.
**Purpose of data collection**	The End Stage Renal Disease (ESRD) Networks serve as a liaison between dialysis and kidney transplantation providers and the Centers for Medicare and Medicaid Services (CMS) that serve as the primary payor for these services. This legislation and subsequent regulations also established health and safety standards applicable to providers of ESRD services and required the establishment of ESRD Network Coordinating Councils. Networks serve as liaisons between the federal government and the providers of ESRD services. The ESRD networks are responsible for ensuring the most efficient use of Medicare dollars for dialysis treatment and kidney transplantation through monitoring quality of care indicators and maintaining timely, complete data on the ESRD program.
**Periodicity**	ESRD Network data are made available on an annual basis.
**Data access**	The ESRD networks database maintains information on over 276,000 dialysis patients, reports can be generated illustrating demographics, patient location, diagnoses, treatment modality, transplantation, and cause of death. This information is published annually by each Network, and special data requests can be filled at any time during the year. Please contact each Network individually when requesting data. A list of the 18 ESRD networks and contact information can be found at: http://www.esrdnetworks.org/Network%20List.htm.
**Data privacy**	All researchers interested in analyzing ESRD network data must submit a proposal that describes legitimate biomedical research that requires the ESRD network data. In addition, researchers must sign the Network's Agreement for Release of Data, which indicates that they agree to comply with restrictions for data use. An example of the data use policy from one of the networks (Network 6) can be found at: http://www.esrdnetwork6.org/data/NetworkProtocol.pdf.
**Statistical and analytical issues**	Data obtained for any single renal network will include only information about persons who have had dialysis within the states that make up that network. Similarly, data obtained for a specific state will include only information about persons who have had dialysis within that state. National data provides information about all persons who have received dialysis, whether or not they received the treatment within their state of residence.
**Data summary**	• Network Annual Report Summaries view website*
**Indicators that can be measured using this Data Source**	Incidence of End-Stage Renal Disease Attributed to Diabetes, Prevalence of End-Stage Renal Disease
**Comments**	View<br>Add

* Links to non-Federal organizations are provided solely as a service to our users. This link does not constitute an endorsement of this organization by CDC or the Federal Government, and none should be inferred. The CDC is not responsible for the content of the individual organization Web pages found at this link.
