# Supplementary material for: An Innovative Approach to Enhancing the Surveillance Capacity of State-based Diabetes Prevention and Control Programs: The Diabetes Indicators and Data Sources Internet Tool (DIDIT)
Source: Prev Chronic Dis. 2005 Jun 15;2(3):A14. (PMC1364523)
Supplement: Supplementary file 5 — View a full-size PDF of Figure 5 (92K) [file 04_0126_05.pdf]

# Diabetes Indicators and Data Source Internet Tool



## Behavioral Risk Factor Surveillance System (BRFSS) - A1c Test

On this page:
Indicator specific information | General information
Definitions | Printer-Friendly Format

| | |
|---|---|
| **Indicator Specific Information** | |
| **Numerator** | Number of persons age 18 or older with diabetes and report that a doctor, nurse, or other health professional has checked the respondent's glycosylated hemoglobin (HbA1c) two or more times in the past year. |
| **Numerator specifications** | Survey question: A test for hemoglobin 'A one C' measures the average level of blood sugar over the past three months. About how many times in the past 12 months has a doctor, nurse, or other health professional checked you for hemoglobin 'A one C'? |
| **Changes to numerator** | Measure changed in 2000 BRFSS (see Historical Questions*) |
| **Denominator** | Number of persons age 18 or older who report that they have ever been diagnosed with diabetes and responded to the A1c test survey question, excluding those diagnosed only during pregnancy. Missing values, refusals, and responses of "don't know" are not included in the denominator. |
| **Denominator specifications** | Survey question: Have you ever been told by a doctor that you have diabetes? (If female, add, Was this only when you were pregnant?) |
| **Technical notes** | |
| **General Information - BRFSS** | |
| **Category** | National and State Data Source |
| **Level of geographic aggregation** | Possible units of analysis include the US and states, territories, and counties that are contained within the US. (Prior to 1996, data were available for only select states and counties.) |
| **Population** | Non-institutionalized adult US population age 18 years or older |
| **Method of data collection** | BRFSS data are collected via telephone survey; most areas use computer-assisted telephone interviewing (CATI). CATI is a system that randomly generates telephone numbers. |
| **Purpose of data collection** | CDC coordinates the collection of BRFSS data for state-level surveillance of health risks among Americans to inform efforts to decrease morbidity and mortality from chronic diseases, injuries, and preventable infectious diseases. Additional information about the purpose of the BRFSS can be found at http://www2.cdc.gov/nccdphp/brfss2/training_ov/default.htm*. |
| **Periodicity** | Data for selected core questions are collected each year, whereas other questions on the core BRFSS are asked on alternative (either even or odd numbered) years. Additional data are collected using various survey modules that supplement the core questionnaire; these modules are fielded on as needed basis, at the discretion of each state. For example, states may include modules for 2 or 3 consecutive years in order to obtain sufficient sample size for reliable estimates. |
| **Data access** | BRFSS coordinators in each state can provide information about data access, BRFSS data, and summaries of results from the BRFSS. A list of all BRFSS state coordinators can be found at http://www2.cdc.gov/nccdphp/brfss2/coordinator.asp*. BRFSS survey data, starting with results from the 1996 survey, can also be obtained from the following website: http://www.cdc.gov/brfss/ti-surveydata2001.htm#survey*. Selected queries of BRFSS data can be conducted at http://apps.nccd.cdc.gov/brfss/index.asp*. |
| **Data privacy** | No personal identifiers are collected in the BRFSS. In addition, so that respondents cannot be identified based on demographic responses, the county code is removed from the public data set for any county with less than 50 respondents. |
| **Statistical and analytical issues** | Data are limited to adults 18 and over. Not all states have the capacity to conduct interviews in languages other than English; therefore, some non-English speaking minorities may be underrepresented. Interviews are limited to non-institutionalized adults; therefore people in nursing homes, prisons, and other residential facilities are not included. Weighting procedures are used to adjust for differences in probability of selection, non-response, and telephone non-coverage and may correct bias introduced by including only persons with telephones. In addition, data are weighted to reflect the age and sex distribution of each state. Detailed information about data limitations, weighting, and other statistical issues can be found in the document "Comparability of Data" that is available at http://www.cdc.gov/brfss/ti-surveydata2001.htm#survey*. Additional information about using BRFSS data can be found in the BRFSS users guide*. States have the option, but are not required, to collect additional data using various survey modules (e.g., Diabetes module). The diabetes prevalence question is in the core questionnaire. The diabetes module, however, is considered optional and there is no guarantee that all states include this module annually. |
| **Data summary** | • BRFSS Trends Data view website<br>• Links to CDC and state publications using BRFSS data describing results for each survey starting in 1996 are available at: http://www.cdc.gov/brfss/pubrfdat.htm*.<br>• Mokdad AH, et. al. (2003). Prevalence of obesity, diabetes, and obesity-related health risk factors, 2001. JAMA, 289(1):76-79.<br>• Mukhtar Q, et. al. (2003). Use of data from the Behavioral Risk Factor Surveillance System optional diabetes module by states. Journal of Public Health Management and Practice, November(Suppl): S52-S55.<br>• National Center for Chronic Disease Prevention and Health Promotion. (2003). Selected Metropolitan/Micropolitan Area Risk Trends (SMART) BRFSS. (Website last updated November 19, 2003). view website |
| **Indicators that can be measured using this Data Source** | A1c Test, Aspirin Therapy, Cholesterol Tested, Dental Exam, Diabetes Care Related Office Visit to a Health Professional, Diabetes Education, Dilated Eye Exam, Flu Vaccination, Foot Exam, Obesity - Primary Prevention in Adults, Obesity - Secondary Prevention in Adults with Diabetes, Overweight or Obese - Secondary Prevention in Adults with Diabetes, Overweight or Obese- Primary Prevention in Adults, Pneumococcal Vaccination, Prevalence of Cardiovascular Complications among Persons with Diabetes, Prevalence of Diabetes in Adults, Prevalence of Diabetic Retinopathy, Prevalence of Foot Ulcers, Regular Physical Activity - Primary Prevention in Adults, Regular Physical Activity - Secondary Prevention in Adults with Diabetes, Self-Blood Glucose Monitoring, Smoking - Primary Prevention, Smoking - Secondary Prevention in Adults with Diabetes, Unhealthy Days among Adults with Diabetes, Visual Foot Exam (self or someone other than health professional) |
| **Comments** | View<br>Add |

* Links to non-Federal organizations are provided solely as a service to our users. This link does not constitute an endorsement of this organization by CDC or the Federal Government, and none should be inferred. The CDC is not responsible for the content of the individual organization Web pages found at this link.
