# Supplementary material for: An Innovative Approach to Enhancing the Surveillance Capacity of State-based Diabetes Prevention and Control Programs: The Diabetes Indicators and Data Sources Internet Tool (DIDIT)
Source: Prev Chronic Dis. 2005 Jun 15;2(3):A14. (PMC1364523)
Supplement: Supplementary file 6 — View a full-size PDF of Figure 6 (44K) [file 04_0126_06.pdf]

Diabetes Indicators and Data Source Internet Tool

End-Stage Renal Disease Networks - Incidence of End-Stage Renal Disease Attributed to Diabetes

On this page:

Indicator specific information | General information Definitions | Printer-Friendly Format

Indicator Specific Information	
**Numerator**	Number of U.S. citizens who have an initial claim for either renal dialysis or renal transplant with diabetes listed as the primary cause of disease during a calendar year.
**Numerator specifications**	Using the ESRD network data, identify persons with an encounter for either renal dialysis or renal transplant due to diabetes during the year of interest. Note that ESRD patients who do not receive either renal dialysis or kidney transplant will not be captured.
**Denominator**	Total resident population for the year of interest (standardized by the direct method to the estimated Year 2000 U.S. population) at a specific point in time.
**Denominator specifications**	Using U.S. census data, determine the total resident population for the year of interest (standardized by the direct method to the estimated Year 2000 U.S. population) at a specific point in time (e.g., mid-year = July 1, end-year December 1).
**Technical notes**	Estimates of prevalence and incidence of ESRD obtained from the USRDS may differ from those provided by the ESRD Networks due to differences in the way data are reported by each group. Specifically, for a given state, the networks report ESRD treatment of residents who received this treatment in-state, whereas the USRDS reports treatment of residents regardless of the location of treatment.
**General Information - End-Stage Renal Disease Networks***	
**Category**	National and State Data Source
**Level of geographic**	Possible units of analysis include the US and states, renal network-defined regions, and counties that are contained within the US. Other geographic
