# Supplementary material for: An Innovative Approach to Enhancing the Surveillance Capacity of State-based Diabetes Prevention and Control Programs: The Diabetes Indicators and Data Sources Internet Tool (DIDIT)
Source: Prev Chronic Dis. 2005 Jun 15;2(3):A14. (PMC1364523)
Supplement: Supplementary file 7 — View a full-size PDF of Figure 7 (183K) [file 04_0126_07.pdf]

Diabetes Indicators and Data Source Internet Tool

At a Glance: Indicators and Associated Data Sources

Filter by: View All Indicators Go

Printer-Friendly Format

View All Indicators

#	Indicator	National Data Source	State Data Source	DPCP-Specific Data Source
1	A1c Level	• HEDIS<br>• NHANES	• HEDIS	• Enhanced Hybrid HEDIS data
2	A1c Test	• BRFSS<br>• HEDIS<br>• Medicaid<br>• Medicare	• BRFSS<br>• HEDIS<br>• Medicaid<br>• Medicare	• California Health Interview Survey (CHIS)<br>• Enhanced Hybrid HEDIS data
3	Aspirin Therapy	• BRFSS	• BRFSS	• California Health Interview Survey (CHIS)
4	Blood Pressure Level	• NHANES		
5	Cardiovascular Death in Persons with Diabetes	• NVSS	• NVSS	
6	Cholesterol Tested	• BRFSS<br>• HEDIS<br>• Medicaid<br>• Medicare<br>• NHANES<br>• NHIS	• BRFSS<br>• HEDIS<br>• Medicaid<br>• Medicare	• Enhanced Hybrid HEDIS data
7	Dental Exam	• BRFSS<br>• NHANES<br>• NHIS	• BRFSS	• California Health Interview Survey (CHIS)
8	Diabetes Care Related Office Visit to Your Health Professional	• BRFSS	• BRFSS	
9	Diabetes Education	• BRFSS<br>• NHIS	• BRFSS	
10	Diabetes-related Hospitalizations	• Medicaid<br>• Medicare<br>• NHDS	• Medicaid<br>• Medicare<br>• SID	• California Health Interview Survey (CHIS)
11	Diabetes-related Mortality	• NVSS	• NVSS	
12	Dilated Eye Exam	• BRFSS<br>• HEDIS<br>• Medicaid<br>• Medicare	• BRFSS<br>• HEDIS<br>• Medicaid<br>• Medicare	• Enhanced Hybrid HEDIS data
13	Flu Vaccination	• BRFSS<br>• Medicaid<br>• Medicare<br>• NHIS	• BRFSS<br>• Medicaid<br>• Medicare	• California Health Interview Survey (CHIS)
14	Foot Exam	• BRFSS	• BRFSS	• California Health Interview Survey (CHIS)
15	Hospitalization for Cardiovascular Disease among Persons with Diabetes	• Medicaid<br>• Medicare<br>• NHDS	• Medicaid<br>• Medicare<br>• SID	• California Health Interview Survey (CHIS)
16	Hospitalization for Lower Extremity Amputations	• Medicaid<br>• Medicare<br>• NHDS	• Medicaid<br>• Medicare<br>• SID	
17	Incidence of End-Stage Renal Disease Attributed to Diabetes	• End-Stage Renal Disease Networks<br>• USRDS	• End-Stage Renal Disease Networks<br>• USRDS	
18	Incidence of Gestational Diabetes	• Medicaid<br>• NVSS<br>• PRAMS	• Medicaid<br>• NVSS<br>• PRAMS	
19	LDL-C Level	• HEDIS<br>• NHANES	• HEDIS	• Enhanced Hybrid HEDIS data
20	Monitoring for Diabetic Nephropathy	• HEDIS	• HEDIS	• Enhanced Hybrid HEDIS data
21	Obesity - Primary Prevention in Adults	• BRFSS<br>• NHANES<br>• NHIS	• BRFSS	• California Health Interview Survey (CHIS)
22	Obesity - Secondary Prevention in Adults with Diabetes	• BRFSS<br>• NHANES<br>• NHIS	• BRFSS	• California Health Interview Survey (CHIS)
23	Overweight or Obese - Secondary Prevention in Adults with Diabetes	• BRFSS<br>• NHANES<br>• NHIS	• BRFSS	• California Health Interview Survey (CHIS)
24	Overweight or Obese- Primary [illegible]	[illegible]	[illegible]	[illegible]
