# Supplementary material for: An Innovative Approach to Enhancing the Surveillance Capacity of State-based Diabetes Prevention and Control Programs: The Diabetes Indicators and Data Sources Internet Tool (DIDIT)
Source: Prev Chronic Dis. 2005 Jun 15;2(3):A14. (PMC1364523)
Supplement: Supplementary file 8 — View a full-size PDF of Figure 8 (65K) [file 04_0126_08.pdf]

National Center for Chronic Disease Prevention and Health Promotion

Diabetes Indicators and Data Source Internet Tool

DIDIT
- Home
- Search
- Reports

EPI RESOURCES

Indicators
- View All
- Browse by Category
- At a Glance

Data Sources
- View All
- Browse by Category
- At a Glance

ABOUT INDICATORS

Background
Bibliography
Glossary
Links
Contact Information

Home »

At a Glance: Data Sources and Associated Indicators

Filter by: View All Data Sources Go

Printer-Friendly Format

View All Data Sources

#	Data Source	Related Indicators	Type of Data Source
1	BRFSS	• A1c Test<br>• Aspirin Therapy<br>• Cholesterol Tested<br>• Dental Exam<br>• Diabetes Care Related Office Visit to Your Health Professional<br>• Diabetes Education<br>• Dilated Eye Exam<br>• Flu Vaccination<br>• Foot Exam<br>• Obesity - Primary Prevention in Adults<br>• Obesity - Secondary Prevention in Adults with Diabetes<br>• Overweight or Obese - Secondary Prevention in Adults with Diabetes<br>• Overweight or Obese- Primary Prevention in Adults<br>• Pneumococcal Vaccination<br>• Prevalence of Cardiovascular Complications among Persons with Diabetes<br>• Prevalence of Diabetes in Adults<br>• Prevalence of Diabetic Retinopathy<br>• Prevalence of Foot Ulcers<br>• Regular Physical Activity - Primary Prevention in Adults<br>• Regular Physical Activity - Secondary Prevention in Adults with Diabetes<br>• Self-Blood Glucose Monitoring<br>• Smoking - Primary Prevention<br>• Smoking - Secondary Prevention in Adults with Diabetes<br>• Unhealthy Days among Adults with Diabetes<br>• Visual Foot Exam (self or someone other than health professional)	National and State
2	California Health Interview Survey (CHIS)	• A1c Test<br>• Aspirin Therapy<br>• Dental Exam<br>• Diabetes-related Hospitalizations<br>• Flu Vaccination<br>• Foot Exam<br>• Hospitalization for Cardiovascular Disease among Persons with Diabetes<br>• Obesity - Primary Prevention in Adults<br>• Obesity - Secondary Prevention in Adults with Diabetes<br>• Overweight or Obese - Secondary Prevention in Adults with Diabetes<br>• Overweight or Obese- Primary Prevention in Adults<br>• Prevalence of Diabetes in Adults<br>• Prevalence of Diabetes in Children<br>• Regular Physical Activity - Primary Prevention in Adults<br>• Regular Physical Activity - Secondary Prevention in Adults with Diabetes<br>• Smoking - Primary Prevention<br>• Smoking - Secondary Prevention in Adults with Diabetes	DPCP-Specific
3	End-Stage Renal Disease Networks	• Incidence of End-Stage Renal Disease Attributed to Diabetes<br>• Prevalence of End-Stage Renal Disease	National and State
